# Mycelial Beehives of HIVEOPOLIS: Designing and Building Therapeutic Inner Nest Environments for Honeybees

**DOI:** 10.3390/biomimetics7020075

**Published:** 2022-06-07

**Authors:** Asya Ilgun, Thomas Schmickl

**Affiliations:** Artificial Life Laboratory, Institute of Biology, University of Graz, 8010 Graz, Austria; thomas.schmickl@uni-graz.at

**Keywords:** biohybrid architecture, bio fabrication, living architecture, beehive, 3D printing, mycelium materials, symbiosis, multispecies architecture, healthy materials

## Abstract

The perceptions and definitions of healthy indoor environments have changed significantly throughout architectural history. Today, molecular biology teaches us that microbes play important roles in human health, and that isolation from them puts not only us but also other inhabitants of urban landscapes, at risk. In order to provide an environment that makes honeybees more resilient to environmental changes, we aim for combining the thermal insulation functionality of mycelium materials with bioactive therapeutic properties within beehive constructions. By identifying mycelial fungi’s interactions with nest-related materials, using digital methods to design a hive structure, and engaging in additive manufacturing, we were able to develop a set of methods for designing and fabricating a fully grown hive. We propose two digital methods for modelling 3D scaffolds for micro-super organism co-occupation scenarios: “variable-offset” and “iterative-subtraction”, followed by two inoculation methods for the biofabrication of scaffolded fungal composites. The HIVEOPOLIS project aims to diversify and complexify urban ecological niches to make them more resilient to future game changers such as climate change. The combined functions of mycelium materials have the potential to provide a therapeutic environment for honeybees and, potentially, humans in the future.

## 1. Introduction

The characteristics that define a healthy environment have changed significantly over human history. The Miasma Theory (400 BC), an obsolete scientific theory, suggested that diseases are caused by bad air [1]. Later, the discovery of germs [2,3] as the origin of diseases led to a public perception of all microbes as pathogenic. As a result, humans’ indoor lifestyle and their yearning for hygiene have set the goals for the design of buildings, and criteria for the selection of materials for isolation. In addition, human activities, e.g., monocultures and intensified agriculture, caused the isolation of other living species from their coevolved symbionts, e.g., through habitat fragmentation. A significant “dewilding” activity is the exploitative domestication of wild animals, especially keystone species like honeybees [4]. 

Among other pollinators, honeybees play a crucial role in conserving biodiversity in flora and fauna. Besides that, they ensure food diversity for human society. Commercial honeybees used in agriculture are affected by a rich spectrum of stressors, such as overcrowded positioning or long-range transportation of hives, and agriculturally applied chemicals. Honey and other bee products crucial to honeybees’ wellbeing are also removed from honeybee colonies. In the meanwhile, cities are becoming megacities due to human population growth. This comes at the expense of diminishing forests, marshlands, and other ecologically vital habitats. Finally, several essential habitat types are decreasing, directly and indirectly harming all bee species. All of these interventions have a significant impact on honeybees’ indoor lifestyle by limiting the diversity of microorganisms within hive interiors and, as a result, the likelihood of symbiotic connections occurring.

Symbiosis is a commonplace relationship of microbial and host elements working together to ensure good nutrition, health, and resistance [5]. When it comes to living in harmony with microorganisms, social insects are excellent models [6]. Among many microorganisms, fungi are one of the key actors within the insect microbiota. Fungi and insects have coevolved a wide array of functional interactions over the past 400 million years [7]. Fungal volatiles, which play an important role in terrestrial ecosystems, also affect the nesting and reproductive behaviour of insects [8,9]. Many termite species actively cultivate fungus to digest the foraged organic materials that they cannot digest themselves internally. The fungus then becomes digestible food for the insects. For this natural form of agriculture, they also build specific rooms in their nests, where the fungus can grow and thrive, forming a prominent form of fungus–insect symbiosis [10]. Humans do not interact with their built environment as effectively as other social animals do. Humans’ architectural product is one that provides some degree of isolation and protection from the “outside” world. It artificially frames human activity [11]. While in other ecosystems, habitats which can support the social lifestyle of animals are inherently permeable to the outside world. Still, the contemporary bio-inclusive architectures propose mechanisms to incorporate other lifeforms into the outer layers of building boundaries. This, once again, separates the wildlife from the human population inside living spaces and does not support the potential health benefits the other life forms have on indoor city dwellers [12,13,14]. Modern beehives are an example of manmade design thinking being imposed on the habitats of other living animals. In general, artificial habitats, such as beehives and human houses, pose challenges in designing how forms, spatial configurations, and materials can affect the microbial diversity that establishes itself in those artificial environments.

### 1.1. Honeybee/Hive as a Model Organism/Habitat

Honeybees are a semi-domesticated animal species: there still are wild colonies thriving in forests or urban areas without human interference [15]. Their natural nests are mostly found in tree cavities that differ from the artificially made habitats concerning their microclimates and microbiomes. Cavities that are large enough for honeybee colony preference (25 L to 40 L), have potentially been formed, thus being occupied by a variety of species for hundreds of years. Such cavities are incrementally formed by various types of other organisms. Mostly initiated with wood rot fungi, other micro and macro-organisms such as invertebrates continue colonising depending on the microclimatic conditions established as a result of the location and sizes of the cavities [16]. On top of pre-existing multispecies communities in these cavities, honeybee colonies continually add diverse types of organic and inorganic materials by collecting particles. These particles range from the size of fungal spores, pollen grains, road and coal dust, and sawdust, up to dead wood [17]. Chlorella alga is one example of an organic material beneficial to honeybees, which they bring to their nest, and which provides nutritional benefits. Researchers discovered that when colonies had access to foraging lands with this alga, they produced more honey than when they moved to a location without it [18]. Forager bees have also been found digging in soil and cattle dung [19] but the reason—if there is one—has not been identified yet.

Honeybee colonies exhibit community-level immunity that also includes the nest material which is essentially a microhabitat for their beneficial symbionts. Resin use and propolis provide self-medication for honeybee colonies with their antiviral, antibacterial, and antifungal properties [20]. Propolis is collected plant resins, mixed with saliva, and wax that the bees use to coat the inner nest surfaces [21]. Beeswax in honeybee nests does not support microbial growth. However, it can be a bioindicator of environmental toxins and colony health: particulates such as larval faeces, shed exuviae, lipophilic chemicals and environmental toxins have been found in beeswax [22]. Pollen is generally the medium in which foragers bring nutrition but also environmental hazards (chemical and biological) to their nests. Additionally, studies have shown that mycotoxin-producing moulds and yeasts that ferment pollen into digestible bee bread, thrive in the conditions in which pollen is stored in the hive [23,24]. Findings on such microbial dependencies advise against the indiscriminate use of pesticides in agriculture. In the case of honeybee colonies, even if the substances do not affect bees directly, they still may be harmful to the microorganisms they essentially need to thrive. It is important to note that determining the microbial communities and their interactions with other organisms or other communities is a complex ecological and technological study.

**Figure 1:**
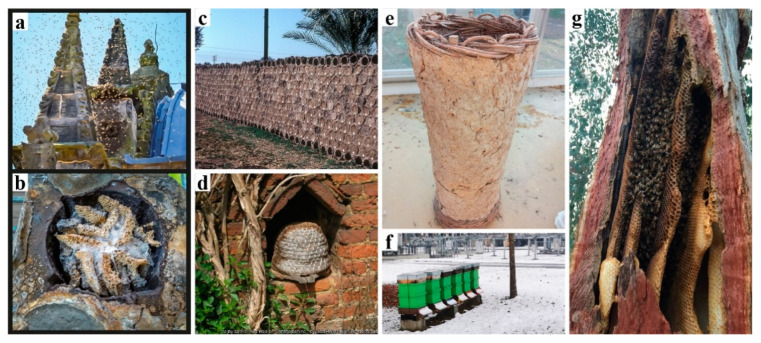
Diversity of man-made beehives and a natural honeybee nest. (**a**) Mediaeval bee haven found in Rosslyn Chapel, Scotland. The north-facing side of the pinnacle when bees are returning to their haven in 2015. (**b**) Fossilised bees’ nest within the pinnacle of Rosslyn Chapel. Photo Credit: Rosslyn Chapel Trust. (**c**) An apiary of stacked mud hives in central Egypt. Photo Credit: Gene Kritsky. (**d**) Bee bole embedded in a historic cottage, UK. cc-by-sa/2.0 by Oast House Archive. Source: https://www.geograph.org.uk/photo/1296874 (accessed on 8 February 2022). (**e**) A handwoven basket hive, coated with ash and cobb, photo taken Cine Beekeeping Museum, Aydin, Turkey. (**f**) Urban beehives near an industrial area, 2021, Graz, Austria. (**g**) A feral honeybee nests. Source: https://forum.canberrabees.com/t/mount-taylor-act-wild-feral-bee-hive-in-a-tree/309 (accessed on 8 February 2022).

Honeybee nests differ significantly from other social insect nests, as the only adaptability found in bees’ nest building is the way they fill pre-given cavities with their combs. They cannot (re-)shape the cavity itself. In contrast to that, termites and ants can dynamically alter their nest enclosures. Still, honeybees can actively regulate the climate and maintain homeostasis within their “prefabricated” nest enclosures. They fill the cavity adaptively as an efficient movement and storage platform for specific behaviour such as fanning, heating, clustering, etc. [25]. Inhabiting a structured but rather static nest topology requires high connectivity between different functional areas, for example efficiently connecting transport paths between the nectar handover area—in the front the near the entrance—and the honey storage area in the back of the hive. The duration of nectar-storing trips between these areas is one of the key regulatory types of feedback of honeybee foraging, ultimately affecting the colony’s pollination activity [26,27,28]. This is just one out of many prerequisites to be considered in a colony’s “hive architecture”. The ventilation and gas exchange should support a fluent transition from a colony’s foraging season mode to the colony’s winter mode and vice versa. However, again, honeybees are highly adaptive and resilient, they flourish in a variety of habitats given the warmth and darkness. Nature-inspired designs imitating or literally being tree trunks have been adopted by beekeepers and designers throughout history, employing a broad range of regionally and organically derived materials (Figure 1a–e). Belgian artist Annemarie Maes uses microorganisms, digital design, and fabrication to connect advanced technology with a living biosystem such as a beehive [29]. There are also projects that use mycelium-grown materials as part of beehive enclosures, but these ideas have never been tested against the risks of long-term colony habitation [30,31].

### 1.2. Fungal Biofabrication

In nature, fungi take the role of primary microbial decomposers, meaning they decompose material in the world’s ecosystems. However, fungi can also be used not only to decompose but also to compose new structures. Mycelium is the vegetative growth of filamentous fungi that bonds organic matter through a network of hyphal microfilaments and it is currently a competitor of several synthetic materials [32]. There are two main types of mycelium-based materials: pure mycelium materials and mycelium-based composites. Pure mycelium materials are generally used and studied for smaller-scale applications such as paper or textile making [33,34] and biomedical applications such as wound healing [35] and tissue engineering [36]. Mycelium-based composites are made by growing the mycelium homogeneously in and around organic waste materials and are generally used for mesoscale modular applications such as bricks [37], thermal insulation, or acoustic panels [38,39], and low-value materials such as packaging [40]. Mycelium materials can be compared to one of the oldest types of composite materials, cob. Cob has organic fibrous material reinforcement, such as straw, which is bound by subsoil. In mycelium composites, instead of the extracted soil, mycelial hyphae are the fully organic and naturally grown binders. This means, if not treated additionally, the whole yielding material is organic. One can also grow these materials with zero waste and tune them to be mechanically intact, thermally insulative and 100% biodegradable. These properties highly depend on the growth factors including the type of lignocellulosic substrate base selected, fungal strains, and climatic control of the growth medium [41]. On the downside, an increased natural degradability means faster degradation or decay, a feature that is usually avoided in traditional building materials. Thus, the use of these materials demands solving additional challenges, e.g., repair or replacement regimes, or finding specific use cases, where such dynamics are not detrimental or even desired. 

During the degradation processes, mycelial fungi’s metabolic activities lead to self-healing [42], beneficial volatile production, and detoxification. However, especially inside buildings with no sufficient natural air ventilation and humidity control, competing bacteria and fungi can harm the mechanical structure of the mycelium. Additionally, mycelium can produce mycotoxins and sporulate which can harm its co-inhabitants. As a result, prior to use, mycelium biofabrication methods and design application scenarios include desiccation of the mycelium biomass.

To date, several biofabrication technologies have been developed to achieve desired shapes and functionalities in mycelium materials. To visually measure growth, the simplest, least complicated, and most generally used approach is to cast a mixture of organic substrate and mycelium inoculum into premade moulds, usually translucent plastic enclosures [43]. This method works effectively for specific design scales where the end product can be grown uniformly. Biofabrication techniques, on the other hand, that specify and generate mycelia’s growth boundaries using computational design and digital fabrication tools, can allow for local variation in material qualities and result in more complex geometries. Textiles can be used to define stay-in scaffolds for mycelium-based composites [44]. Textile logics can be translated into the filament scale, such as the structural stay-in scaffolds produced using the Kagome weaving method in the FUNGAR Project’s building elements [45]. In another recent work, computationally generated scaffold morphologies have been 3D printed and inoculated via a robot arm equipped with sensors [46]. Furthermore, researchers and designers have been successful in 3D printing pre-inoculated viscous materials directly [47,48].

### 1.3. A Therapeutic Design Problem

In this article, we propose a hybrid construction method for building more bioreceptive and bioactive beehives using living fungal mycelia formed via 3D printed stay-in scaffolds. Our main goal is to combine the thermally insulative properties of mycelia with its medicinal, potentially microbiome modulating properties. We use parametric design tools and fused deposition manufacturing to produce these mycelium scaffolds. Our hive morphologies are designed aiming at honeybee colonies that self-organise similar to how they reside in hollow trees (Section 3.3) while reducing the energy loss of the hive. We used the quantitative and qualitative aspects of tall and narrow tree cavities as a design reference since the community-level immunity of honeybees has evolved in such environments. The main function of the overall hive morphology is to be durable supporting a full bee colony, living mycelium body and against changing weather conditions. This “therapeutic design problem” is a challenging task in creating and testing artificial habitats. It requires setting up empirical experiments to study one-to-one scale hive designs, to compare morphologies that are successfully occupied long-term (minimum one year) and both by honeybees and mycelia.

The design of therapeutic inner nest environments starts with a bioreceptive strategy for the overall morphology. There are two layers of bioreceptivity to be considered in fungal architectures. First is the receptivity (to mycelia) strategy used in designing the overall morphologies. The overall morphology of the fungal construct is primarily the morphology of the reusable or sacrificial formworks in/on which the mycelia grow. This first layer affects the second layer, which is the receptivity (to any other microorganisms and insects) of the pre-established mycelium arising from variation in surface qualities, or density differentiation throughout mycelial volumes. For functional applications, the second layer of bioreceptivity is minimised when the mycelium is heated and desiccated after its dense network formation. However, our goal with therapeutic mycelial beehives includes both layers of bioreceptive design. First is to enable the release of beneficial fungal compounds towards the hive interior. This is only possible with a living—not necessarily growing—mycelium structurally supported by 3D printed stay-in scaffolds. This approach can be a counteraction to the modern beekeeping sector. The modern beehives fall behind in terms of design characteristics that affect the climate conditions that are most relevant for honeybees and their symbionts. For example, one study shows that tree cavities provide better humidity levels compared to traditional modular box hives [48].

A targeted approach to therapeutic design problems can be about designing with specific and known medicinal properties of some fungi. Recent research shows that fungus species with antiviral and antibacterial compounds modulating microbial communities that are beneficial to humans can also boost the immune system of honeybees against specific viruses and bacteria when fed to the bees [49,50,51]. For embedding such properties in the enclosure material by growing specific fungal strains into hive morphologies the mycelia need to be kept alive during the honeybee colony inhabitation. In a targeted case like this, the challenge is to find a way to match the environmental conditions—temperatures, humidity, pH and oxygen levels—of the fungal habitats with those of the harmful organisms. For instance, a directed evolution strategy is used to breed an entomopathogenic fungus *Metarhizium brunneum.* This fungus is known to inhibit the growth of a honeybee pathogen Varroa Destructor, but naturally lives in lower temperatures, so it is bred to thrive in mostly affected brood areas in the hive (avg. 35 °C) [52]. Genetic modification or breeding of mycelial fungal species are interesting for these applications. In addition to what mycelia can do for honeybees, one example of how honeybee activities might support mycelial life can be the propolis enrichment of habitats. It has been shown that propolis can be used as a growth supplement for decreasing contamination risk in the mushroom production industry [53].

Moreover, novel therapeutic properties of mycelial habitats for honeybees might lay hidden in plain sight, as microorganisms embedded in the construction material may be able to perform certain beneficial support functions for the honeybee collective. However, these properties and abilities may well depend on the environmental conditions they are growing in the microclimate of the hive, which is a special ecological niche for microorganisms, precisely controlled by the bees but still affected by the environmental conditions of the hive surrounding. This creates the main aspect of complexity that apply to the case of fungal biodesign for social animals. A circular feedback loop is in effect here, as the bees control the inner nest climate to a high extent, affecting the microbial communities, which in turn can affect the bees in return. The mycelium hive body with a large number of fungal cells also has an impact on the inner climate when they respire, degrade and regenerate. Even though this might not alter the inner hive climate as much as or as rapidly as the honeybee colony can, it would trigger the highly sensitive bees to more actively regulate the inhive climate in a homeostatic state. This would in turn affect the mycelium’s morphology, molecular composition, and survival.

## 2. Methods

### 2.1. Bio (Material) Coupling

To investigate the interactions between honeybee nest-related materials, honeybee pathogens, and mycelial fungi, we employ methods from classical microbiology in-lab and honeybee behavioural biology on-field. For measuring the therapeutic properties of mycelia, we set up microbiological assays commonly used in insect immunity studies such as the lytic zone assay. These assays are used to measure the ability of any substance to break apart bacterial cell walls. Bioassays are controlled experiments that are commonly used to assess the potency of a bioactive agent—in our case, living or inactive mycelium—as inhibitors of pathogenic microorganism growth in comparison to standard measures. These experiments are typically designed in such a way that the environmental conditions are suitable to the pathogenic matter whose growth the test matter is hypothesised to inhibit. In addition to the inhibition assays, we make Petri dish experiments where a selection of organic nest matter—propolis, wax, pollen, honey, bee bread, etc. —are placed next to a mycelium patch and incubated at temperatures in which the nest materials would exist naturally. For coupling the living bees and our hive material designs, we make field experiments with full sized bee colonies. In these experiments, the prior criterion for the mycelium material is the selected fungus species being non-pathogenic to humans, bees, and plants, as well as to the local ecosystem.

### 2.2. Bio (Scaffolding) Design

We developed a design-to-fabrication methodology (digital design tailored for additive manufacturing) to make structural and nutritional scaffolds in/on which mycelia attach and grow. This method promotes a homogenous and fast growth of the selected fungal matrix (mycelium of fungus species and strain). In our case, we used fused deposition modelling, a sub-caste in the 3D printing family holds forth the promise of “digital craft” [54]: a set of topological and geometrical operations to produce patterns as continuous extrusion paths and overall morphologies which at the end are represented as a set of instructions for the 3D printer.

**Figure 2:**
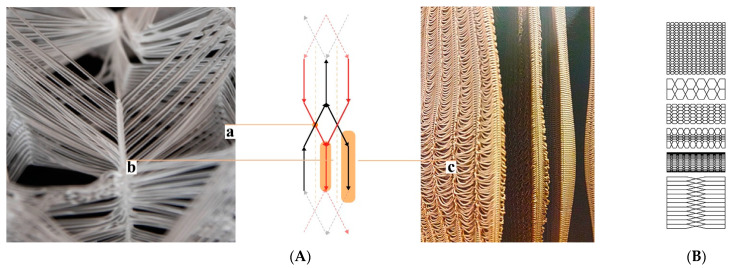
Topological operations for continuous toolpath drawing. (**A**) Hexagonal weaving. (a) Point bonding areas. (b) Linear bonding area. (c) Drape zone. (**B**) Density differentiation via the VO method.

**Topological Operations:** Our current toolpath drawing pattern is based on radial or mesh hexagonal grid topologies. Following a continuous weaving sequence of hexagonal cell control points, one polyline is formed. We call it “continuous weaving” because the toolpath is not interrupted, extrusion is continuous. The deposited material is like a continuous thread (Figure 2).

**Geometrical Operations:** In the first digital method, which we call the “variable-offset (VO)” method, multiple values are used to offset the contour lines of the user-specified geometry (distances), defining the outer boundary as well as the inner material density with consideration of the spaces for mycelium inoculate. The spaces between the offset lines are then intertwined using hexagonal weaving (Figure 2A). The initial geometries can be created with the intuitive top-down control of parameters. From there on a cluster-oriented Genetic Algorithm, using the Biomorpher [55] plug-in for Grasshopper3D [56] rapidly explores a confined design space inside a parametric design model and provides interim 3D representations of design varieties. With an interactive evolution design tool, it is possible to rapidly generate morphological varieties while dealing with competing quantitative factors, such as the sizes of spaces required for the occupation of different scales of organisms and the time/material needed for 3D printing [57,58].

**Figure 3:**
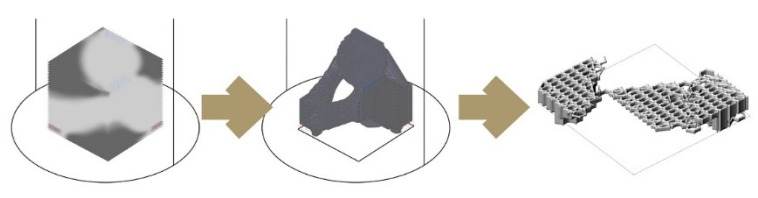
IS method. From left to right: density distribution in voxel space using Millipede, isomesh visualisation, and hexagonal weaving for voxel definition.

The second digital method, “iterative-subtraction (IS)”, allows for the distribution of voxels in 3D space according to specified load and support requirements. This is an iterative optimisation process using finite element functions. Density distribution throughout this voxelised space is defined in accordance with benchmarks, which in our fungal hive case are drawn in consideration of honeybee occupancy loads, spatial layouts for its landing zone, protected spaces or dark zones, and minimisation of material used for production [59]. The voxels are then replaced with hexcells and translated into a toolpath with the hexagonal weaving drawing method (Figure 3). Numerous toolkits can significantly assist in the topology design process in Grasshopper3D, we used Millipede [60]. This mode of operation yields a material microstructure with uniform porosity. So, if needed, geometrical attractors—lines or points—in the parametric hive model can be used to achieve density gradients. We previously presented this application of topology optimisation in a speculative design and construction scenario for multispecies architectural boundaries, the *Co-occupied Boundaries* Project [61].

Using our design approach, we are able to provide more direct lines of communication between the digital geometries, machine parameters and physical model. Finally, we may also adjust both surface and inner porosities of the scaffold based on performance parameters for temperature and humidity control. Because it enables structural design with physics calculation, the IS technique may be more beneficial for designing overall structures capable of housing a beehive colony in an elevated position above the ground-similar to forest habitats in large trees.

### 2.3. Biofabrication

**Figure 4:**
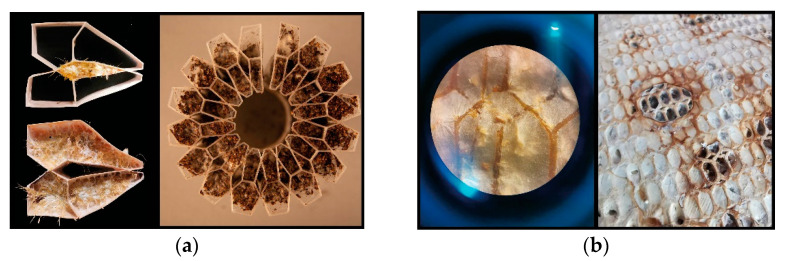
Artefacts produced with different methods. (**a**) “Infill-feed”. (**b**) “Self-feed”.

Depending on the material and microstructure resolution defining the overall morphology, we use different fungal inoculation methods. In what we call the “infill-feed” method, we manually fill the vertical tubular cells with mycelium inoculated solid substrates (Figure 4a). In the second method, the “self-feed” method, the liquid fungal culture is poured directly onto the printed scaffold. In this case, the printed scaffolds should have higher grid resolution and therefore must provide enough nutrition for the mycelia, nutrition providing surface area for mycelium growth (Figure 4b).

## 3. Experiments

### 3.1. Materials

**Figure 5:**
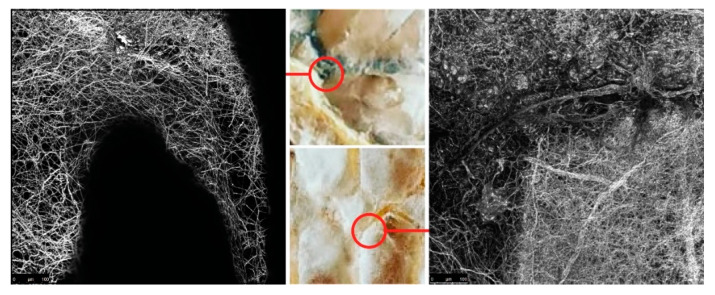
Autofluorescence of fungal hyphae (white) was captured with confocal laser-scanning microscopy and used for visualising the virtual slices of material. Left: TV mycelium and lignin PLA: Right: TV mycelium and GrowLay™.

**Figure 6:**
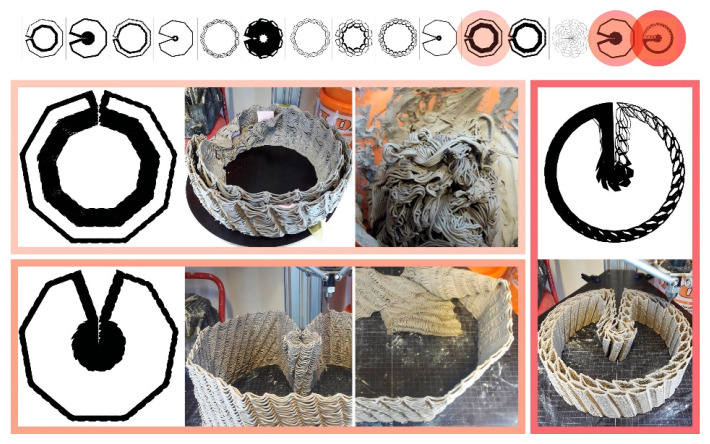
Clay extrusion toolpath tests to find the hex-weaving parameters and overall geometry. The darker red colour shows the final iteration which we used to 3D print the whole clay scaffold.

The surface area of the nutritional matter that a fungus can attach to is an important factor that influences hyphae breakdown of specified material. It can be increased, or controlled, by using extrusion of lignocellulose rich substances. First, we took a preliminary investigation into a wide range of manufacturing parameters that affect the crucial geometrical attributes necessary for the growth of mycelium. In a continuous deposition, variables like print head speed and distance from the previously printed layer greatly impact the thickness of extruded lines, thus the surface area of the organic molecules on which fungal cells can attach. An experimental composite filament (GrowLay™) [62] proved to be the best candidate in our commercially available polymeric material assortment. GrowLay™ is made of cellulose particles, polyvinyl alcohol or PVA which is a water-soluble synthetic polymer and another backbone polymer –that the producer does not prefer sharing. Once the PVA is removed, the printed structures retain microcapillaries and increased surface area of the cellulose. Using the “self-feed” method, we prepared samples with TV hyphae growing on GrowLay™ and *T. Versicolor* (TV) hyphae growing on lignin infused polylactic acid (lignin PLA). In Figure 5, we demonstrated our observation of TV hyphae growth on these two 3D printing materials using microscopic scanning technology. As foreseen, hyphae could grow across the GrowLay™ material (Figure 5), which remains with microcapillaries after the removal of the water-soluble polymer. We also explored paper clay as a stay-in scaffold material [63] and clay extrusion to build. When compared to synthetic and engineered bioplastics, clay printing comes with its own set of challenges due to its organic nature. Therefore, we first experimented with the toolpaths and the 3D printing parameters in order to establish porous boundaries which are able to hold the mycelium substrates and avoid layer collapses (Figure 6). However, we did not make a microscopic scan of clay mycelium samples.

For the large-scale prototypes, we used a large thermoplastic 3D printer, Reprap BIG, using the GrowLay™ filament described above. We refer to them as GrowLay Hive-1 and GrowLay Hive-2. The third and the most recent one was printed with clay, with a liquid deposition modelling printer, Delta WASP 40 100 Clay, and we refer to it as the Mycelial Clay Hive. Both printers were placed in room conditions in springtime without indoor climate control. These hives are described in more detail in Section 3.4.

For measuring the fungal mycelia’s potency as bioactive agents against potential honeybee pathogens, we used two bacterial players. *Micrococcus luteus* is a gram-positive bacterium commonly used in the initial stages of inhibition zone assays in insect immunology studies. For more targeted studies we used Paenibacillus larvae cells—the causative bacterium of deadly American Foulbrood (AFB) disease in honeybee colonies. Both agents were pre-initiated and grown to an active stage in agar Petri dishes. As fungal players in these assays, we used mycelia of five different species: *Trametes versicolor* (TV), *Pleurotus ostreatus* (PO), *Ganoderma lucidum* (GL), *Hericium erinaceus* (HE), and *Grifola frondosa* (GF). However, for the targeted assays we mainly focused on the mycelium of *Trametes versicolor* (TV) which is a common mushroom producing polypore fungus and is commercially known for its anticancer ingredient Krestin (PSK, a protein-bound polysaccharide). It has also been found that Krestin is also a strong antibiotic against microbes pathogenic to humans. This and other medicinal compounds are present in the mycelium of TV. For the large-scale design prototypes, we used PO, GL, and TV mycelia. PO, also known as oyster mushroom, is a widely grown edible mushroom with a rapidly spreading mycelium that efficiently utilises substrate resources, making it a good material maker [64]. GL belongs to the *Ganoderma* species which is broadly studied and showed antiviral properties effective against honeybee deformed wing virus [49]. In general, our biological organism selection criteria were mostly about the honeybee related properties.

### 3.2. In Vitro Coupling

We had a series of laboratory experiments to characterise the antibacterial activity of specific fungal species. First assays showed us the ability of TV mycelium grown on GrowLay™ to lyse bacterial cell walls, based on the lytic plate assay, using freeze-dried cells of *M. luteus*. Following this, we carried out the inhibition assay with living *P. larvae* cells. This is different from lytic activity, in that it shows the ability of the samples to inhibit the proliferation of bacterial cells, leading to the death of the bacterium. However, we had yeast contamination in all our plates potentially due to the incubation temperatures (35 °C), a much higher temperature than what living mycelium needs to grow. As the third testing method, we homogenised the patches of mycelia of different fungal strains. This process breaks down the cell walls of fungi and frees compounds which might be bioactive and antibacterial, and it potentially kills all living agents in the solution. We first cultured the mycelia of TV, PO, GL, HE, and GF. After we scraped 1 cm × 1 cm mycelium patches from the agar cultures, we diluted them in 1 mL PSB (phosphate solubilising bacteria) water. Then, we used a TissueLyser II to break down the fungal cells, for 15 min and shake with maximum frequency. Since the scraped mycelium culture was mixed with the substrate, the solutions were too thick for the micro filtering process which we needed for the last phase. Therefore, we used a centrifugal shaker to have clearer liquids at the end. After we filtered the solutions, we incubated the *M. luteus* cultured media with these solutions in conditions where the *M. luteus* thrive (35 °C). However, this assay did not show us any positive results as opposed to the first assay where we have used a grown and living patch of TV mycelium. 

In unsterile conditions, we placed living TV mycelium and propolis in six malt extract agar dishes. The propolis was collected directly from our hives at the HIVEOPOLIS Honeybee Research Field Laboratory at the Botanical Gardens in Graz, Austria and we did not pre-process it to pasteurise or similar. We did not have any control samples, yet from our experience, agar cultures should be prepared in highly sterile environments to avoid contamination. Yet, the mycelium grew fast and healthy in our dishes with propolis. This can be in favour of the mycelium survival in a living fungal honeybee hive. Then, in a closed plastic container, we placed living TV mycelium grown on a solid substrate together with a wax comb piece, collected from one of our hives and not pre-processed to sterilise. We observed a superficial mycelium coverage on beeswax without degradation of the wax, potentially caused by the fattiness of the wax. We have not performed any coupling assays with pollen, honey, or other substances such as bee bread or royal jelly. Among these, we think that the pollen storage area is a good candidate for the fungal mycelia that we introduced to find nutrition and water. Thus, if our mycelial fungi can survive on pollen as a bio-fungicide, the mycotoxin release can be inhibited in favour of the honeybee colony. This also opens mutualism, in terms of an organic coupling towards therapeutic hives indoors. In terms of specific honeybee bacterial pathogens, we concentrated on AFB disease, which can be detrimental to any colony as fast as three weeks. AFB only attacks the honeybee larvae, located in the brood comb area within the nest with stable temperatures between 32–36 °C. The conditions that AFB and larvae live in, especially the temperature, are different from the conditions of basidiomycetes fungi habitats. However, in the longer term, as directed evolution techniques become more common, we can improve our material-maker fungi to survive in higher temperatures and train them to inhibit the growth of pathogenic bacteria. 

### 3.3. First field Experiment with Living Bees: “Beeocompatibility”

**Figure 7:**
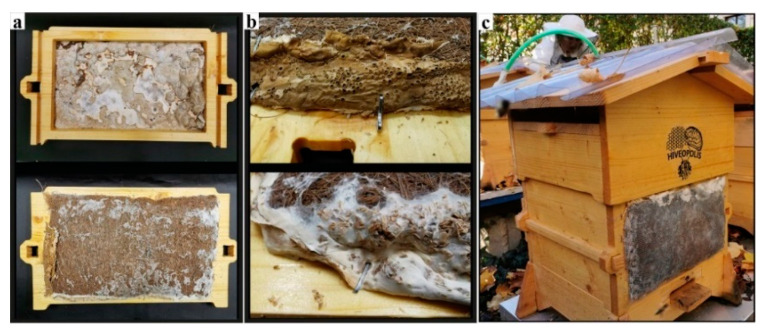
BCN Wárre Hive with mycelium retrofit. (**a**) Retrofit hive wall with GL mycelium grown on coconut fibre mat. (**b**) Fixing details. (**c**) One of the three mycelium retrofit hives.

To test if honeybees are tolerant to mycelium materials, we set up a controlled experiment with living bees. We adapted the natural beekeeping friendly and open-source BCN Wárre Hive design and fabrication models provided by the OSBeehives Project [65]. We selected the mycelium of GL. As growth substrates, we used coconut fibre mats since coconut is hostile to bacterial and yeast contamination and has acidity levels suitable for mycelium (5.5–6.5). The cut-out coconut mats were soaked in water for 12 h, hand pressed, and autoclaved at 121 °C for 20 min. We laid the pieces from a pregrown GL mycelium on the fibre mats, placed them in shallow plastic boxes without lids, enclosed in large plastic bags and then incubated them for 14 days at 23 °C. When we were satisfied with the mycelial colonisation, we dried the pieces in a kitchen oven at 50 °C for 2 h each. We used the mycelial side oriented towards the inside of the hive. We stapled the leatherlike edges on the wooden panels and placed a metal mesh to protect the fibres from being eaten by other animals (Figure 7).

On 20 July 2019, we introduced six small-sized bee colonies (3000–4000 bees each) into each hive, three of them with mycelial walls and three with regular 18 mm thick wooden walls. We monitored the climate within the hives for two months from 26 July to 6 September, with combined temperature and relative humidity sensors, Beebots, provided by one of the HIVEOPOLIS research groups, Pollenity. The ambient temperature and relative humidity measurements were from near mycelium walls in three of the hives, and from the same locations in the control hives. These measurements were essential for tracking the microclimatic differences that occurred near two materials, as well as for our long-term hive design programme where the mycelium remains alive.

This experiment gave us several insights into the honeybee and mycelium material coupling and what to consider in experiment set-ups with hives accommodating full colonies.

- We had many contamination problems during the mycelium wall preparation and learned that the contamination can be avoided only with premeditated clean lab protocols, especially in such experimental scenarios in which the materials must be ready to be tested on-site with strict deadlines. The preparation labour and errors cost us a late start of the whole field experiment.
- Bees chewed out the mycelium from areas that are softer than others. We can only hypothesise about what they did with the mycelium: they might have moved it out from the hive or consumed it. We think that the second possibility could be beneficial for the colony given the nutritional and therapeutic benefits of mycelium.
- Regarding the hive set-up, we realised that the bees should have been blocked from the empty quilt box (a shallow empty volume below the feeder designed to be filled with humidity capturing material). As this area gets warmer and nearer to the feeder, some colonies initiated their nests there, instead of the targeted mycelium and sensor-attached area. The temperature and relative humidity measurements did not provide a clear distinction between the climate within the mycelium retrofit hives and fully wooden hives, potentially because the nesting spots were different in each hive.

Only one of the six hives survived until the following summer season. This survivor hive was one with mycelium attached. We think that the reason is that we populated the hives late in the season, not giving bees enough time to reproduce, collect pollen and nectar, and build wax combs to store enough honey. Additionally, the weather conditions were particularly challenging that year, with heavy rainfall leading to floodings in many adjacent buildings. The colonies and their wax combs were so small that there was too much empty space in the hives before the winter and not enough insulation on the walls.

### 3.4. Design Experiments

#### 3.4.1. Digital Hive Morphologies

**Figure 8:**
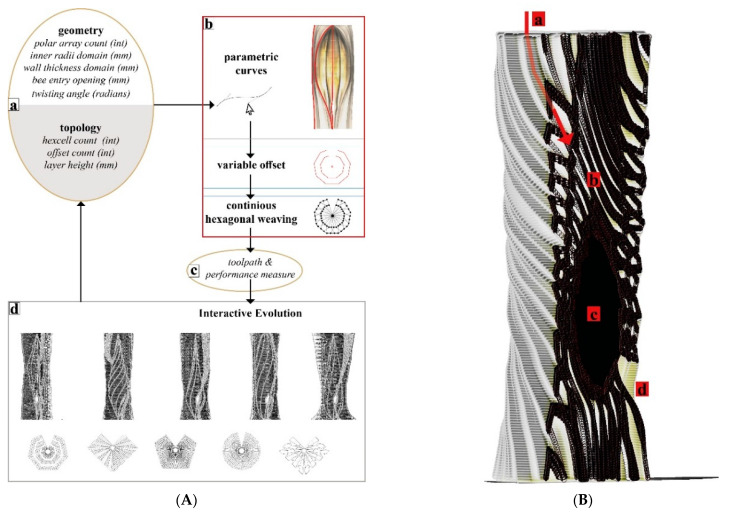
**Design scheme for hive morphologies using VO.** (**A**) Parameters (a) are fed to drawing algorithm (b), according to measurable performance criteria (c), we assess the 3D cluster representatives, (d) and reach a design iteration. (**B**) Output of one design iteration for a stand-alone hive morphology. (a) The tubular hex channel for “infill-feed”. (b) Thick voluminous mycelium roof area for capturing moisture. (c) Warm and dark nest space. (d) Landing platform for the honeybees.

Based on the studies describing the living conditions of honeybees in feral nests [15], we identified our geometrical benchmarks for the overall structure such as the inner nest shape and volume and entrance opening size. Figure 8 demonstrates a design study for generating stand-alone hive morphologies. Load-wise, the inner nest should accommodate approximately 60,000 honeybee workers plus one queen, including their colony’s honey, the wax, and other nest materials, weighing as much as 80 kg in total. This cavity should be well insulated to avoid temperature fluctuations within the nest space. For providing the fungal mycelia’s scaffold structure, a high porosity for oxygen distribution and structural stability—especially during growth by degradation—are necessary. In consequence, the honeybees’ nesting volume should be surrounded by a highly thick and voluminous enclosure of mycelium composite to provide sufficient insulation and mechanical stability. 

**Figure 9:**
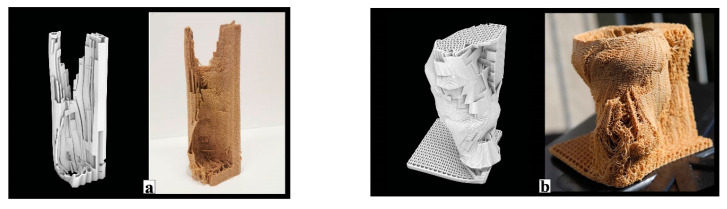
Morphologies created using IS method. (**a**) Open structure for a swarming honeybee colony to nest and 1:5 prototype 3D printed with a wood-infill filament. (**b**) Enclosed structure for a feral honeybee colony to nest and 1:5 prototype 3D printed with a wood-infill filament.

One of the lessons we learned by exposing bees to mycelium-based composites in the first field experiment was that they removed the soft parts of the material. Therefore, our scaffold serves as a barrier between the bees’ nest and the dense yet soft mycelial zones, while allowing hyphal reach. We use honeybees’ natural tree nests as a reference for the inner nest geometry of our one-to-one size mycelial hives (further described in the next section). Figure 9 shows two morphologies created using the IS method, which only takes a basic load and support conditions into account in the topology optimisation part and is then edited to have open (Figure 9a) and enclosed (Figure 9b) bee habitation spaces. We show the 1:5 prototypes because this design method development is directly related to the following 3D printing and self-feed procedures.

#### 3.4.2. Fully Grown Mycelial Hives

Here we describe the full-scale mycelium-based hives produced by using the VO method. From summer 2019 to summer 2021, we produced three types of mycelium-based hives.

**Figure 10:**
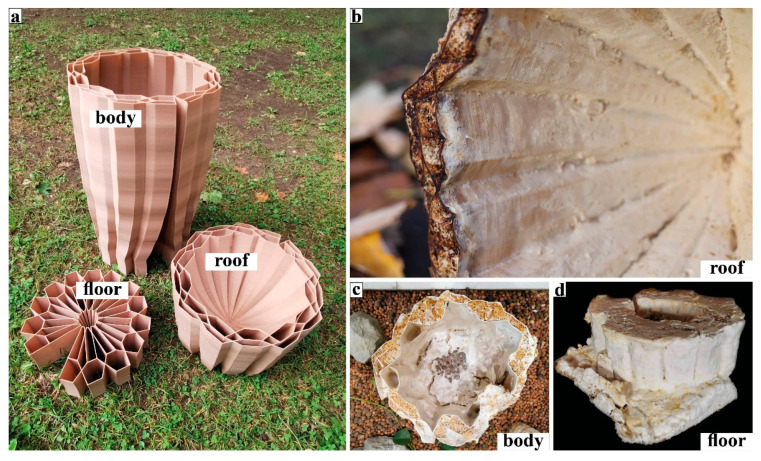
GrowLay Hive-1 (**a**) 3D printed scaffold before the PVA removal. (**b**) A close up picture taken two years after: the powder like mycelium remains on the surface. (**c**) Vienna Design Week, 2019: mycelium kept degrading the hive scaffold and as we misted it, the fruit bodies emerge. (**d**) A picture taken right after the mycelium hive part is removed from its box and placed in the kitchen oven.

In the summer of 2019, our GrowLay Hive-1 was printed in three parts in 32.5 h using approx. 5 kg of filament. We soaked the printed parts in the purified water for 10 h each, refreshing the water every 2–3 h. This was a delicate and laborious process which also caused the thin extrusions to lose their stability, weakening the layer adhesion, and yielding rickety parts with decreased structural scaffolding capacity. After the removal of the PVA, we exposed the parts to ultraviolet light on a clean bench for 12 h (to kill the contaminating microorganisms). After 6 h of drying time on a clean bench, in separate clean plastic boxes, we filled the vertical channels of the three hive parts with grain seeds and beechwood dust inoculated with the PO mycelium. After three weeks of growth at room temperature (23 °C), these modules were taken out of their plastic boxes and left to dry in a kitchen oven for six hours. We moved the largest fresh and growing part to the open exhibition space of the Festival Headquarters, at the Vienna Design Week 2019. We let the hive fruit by exposing it to more oxygen, light and water as part of our exhibition “Biohybrid Superorganisms Diversify Urban Ecological Niches” (Figure 10).

**Figure 11:**
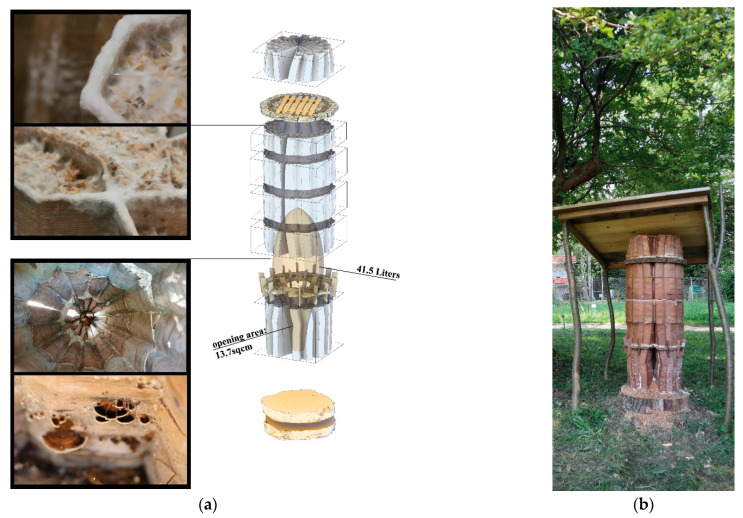
GrowLay Hive-2. (**a**) An exploded axonometric drawing of the whole construction, showing details of the fungal parts. (**b**) Outdoor assembly during late summer 2020.

Within the GrowLay Hive-2—which had a slightly larger, 45 L inner nest volume compared to the GrowLay Hive-1—we aimed to introduce a honeybee colony in the spring of 2020 (Figure 11a). Therefore, the hive’s stability and durability became priorities. Instead of increasing the structural stability via geometry and density of the printed structure, we kept the PVA, the water-soluble polymer, to support the scaffold with better particle adhesion. For the ease of mycelium infill and handling, we printed this hive in six smaller parts compared to the GrowLay Hive-1, in 45 h using approx. 6 kg of filament. The first prototype of GrowLay Hive-2 was disposed of due to *Trichoderma* fungus contamination. The next one could be produced only in mid-July 2020 which is almost the mid of the bee season. This time, the TV mycelium was grown in the vertical channel with birchwood chips. The inoculation process was in a laboratory but not on a clean bench. After 4 weeks of colonisation, beginning of September 2020, we moved the hive under a small wooden protective shelter into an outdoor setting at the HIVEOPOLIS Honeybee Research Field Laboratory. However, it was too late in the season for a honeybee colony to start building their natural comb structures which require a lot of their energy. To avoid risking a foreseeable winter death of such a late-established colony, we left the hive empty (Figure 11b).

The most recent hive scaffold was printed with clay. The overall form of the digital model was built using the VO method and after determining the toolpath drawing parameters. To achieve the required honeybee inhabitation volume within fabrication constraints (max. printing diameter = 40 cm and heights of each module kept to a maximum of 15 cm to avoid layer collapse), the walls of the mid-body parts had to be thinner, making the inoculation—filling the vertical gaps with mycelium inoculated flax fibres—more difficult, if not impossible in those areas. Furthermore, because we initially added water to adjust the viscosity, the printed clay shrank by nearly 20% during the drying and firing processes, as a result, the honeybee inhabitation volume decreased from 39 L to an average of 30 L. The more we expand the inner nest volume, the thicker the mycelial wall should be to maintain the thermal stability within the hive. This would require either dividing the ring-like modules into printable sizes across their cross sections or using a larger 3D printer. Additionally, this would result in longer toolpaths, therefore a larger surface area through which the clay would lose water and higher shrinkage rates.

Our Mycelial Clay Hive is made up of 13 ring modules and took 15 h to print. Before printing the clay scaffold, a commercially available stoneware paper clay was hand mixed with 15% of its weight with tap water to reach a suitable printing viscosity. When compared to the previous GrowLay Hive modules, the clay ones had a significantly higher weight, which was beneficial for overall stability, but it had to be kept to a minimum for subsequent single-person handling. To avoid cracks, the units were air dried for two days after 3D printing, loosely covered with plastic sheets. They were then fired at 1200 °C. We filled the voids in the modules with pregrown mycelium spawn and ground particles of entire flax plants that had been inoculated with TV’s mycelium. The inoculation procedure was carried out as quickly and cleanly as possible without sterile conditions. They were incubated in a 23 °C ambient room temperature for the first ten days. During this time, we used a heat mat connected to a temperature sensor and controller, alternating the plastic boxes. During this time, one module that had cracked during transportation was stabilised along with its constituent mycelium. 

**Figure 12:**
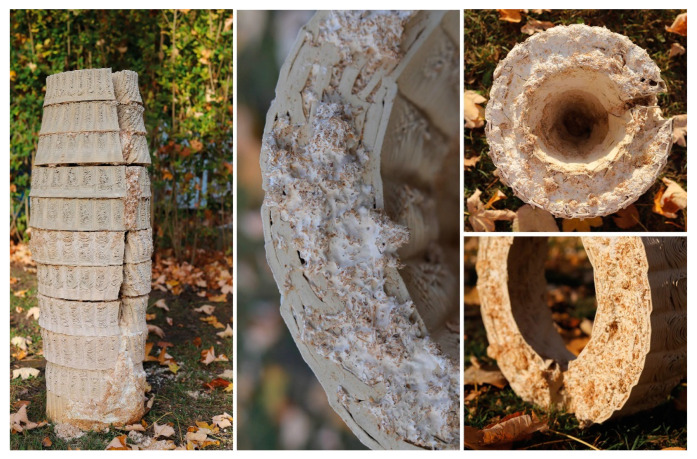
Mycelial Clay Hive.

The main challenge we had in making the first two hives was that the aeration of the mycelium inoculate within the scaffold channels was not sufficient. This created a fast formation of the thick mycelial skin on the surfaces, which were exposed to air while leaving the inner areas of the walls only marginally colonised. When hydrophobia and protection from intruding animals are required, this thick leatherlike differentiation of mycelium can be beneficial. However, when it occurs at the intersecting surfaces of modules that are stacked on top of each other, it inhibits the further hyphal growth for biowelding separate modules together. Another problem was the deformation of the module geometries during the incubation period with high moisture levels. This resulted in an ill-defined continuity in the overall hive geometry. We observed that PVA drips out, creating a strong chemical border that blocks the mycelium from penetrating into the extruded scaffold material. So, if GrowLay™ was a decided material, removing the PVA and improving the structural integrity of the hive via the scaffold design variables would have been a better solution. To compensate for these losses of deformation and stability, we used bamboo sticks as an inner reinforcement when we installed the hive in the garden. The hive was disassembled in Autumn 2021, and we observed that several other animals—such as snails, spiders, and soil insects—already occupied the hive. In the clay scaffolds, the mycelium infill grew faster and more uniformly than in previous GrowLay Hives due to the increased porosity created by the toolpath and the micropores formed after firing the fibres out of the modules. Mycelium’s robust and uniform growth allowed for the biowelding of modules with a large enough surface area in contact (Figure 12).

## 4. Discussion

To investigate the animals’ (including humans’) dependency on diverse microbial communities in their habitats (our built environment), we use the honeybee as a model organism and fungal architectures as a biodiversity maximising strategy. In this paper, we showed our hybrid construction method and a design framework for merging two complex and dynamic material systems—the honeybee superorganism and mycelial networks—in order to reassemble a potentially lost link between the social insects’ wellbeing and bioactive inner nest spaces. We report our findings and insights on the following topics in this paper.

The architecture of honeybee nest enclosures and the architecture of their comb construction differ. Nevertheless, mycelial architecture is comparable to both. The honeybee colony builds a custom comb structure within established nest enclosures. These enclosures function as heat and light barriers, and bees themselves engage as material constituents of this dynamic multi-material system. Thus, the nest’s material components are the bees’ products and also their bodies. Mycelial material systems are similar to honeybee nests in that they are adaptive material systems that are entangled with their surroundings and actively manage their local environment, such as chemical and microbiological conditions, in order to survive. When placed in predetermined nest enclosure formworks or scaffolds, the microscale hyphae span the entire geometry. Like honeybees, mycelium largely remains within the physical domains of its prepared form while being able to adjust its behaviour dynamically. In addition, as mentioned earlier, wood-rotting fungi are already present in the natural tree hollows where robust wild bee colonies live, and mycelium architecture is already present in detectable levels in honeybee habitats and their nearby ecosystems.

We argued that the mycelia have been part of the social immunity of the honeybees while co-occupying the tree cavity nests throughout evolutionary history. However, the concentration of mycelia in the nest enclosure materials is significantly higher in the fungal hives we presented. This could imply that the natural balance of fungal metabolites, honeybee symbionts, and honeybees may not be established in fully grown mycelial hives. Our goal is not to reconstruct a tree cavity, yet we aim to seed sentient material systems in which mycelium takes part and renders it specifically receptive to microbial communities that have coevolved with the honeybee species over millions of years. Nothing exists in isolation in nature, especially in a honeybee hive. The adaptive mechanisms found in living materials arise due to their form, multiresolution microstructures, causing them to behave in nonlinear ways, responding to external stimuli in unpredictable ways. In the targeted microbiology coupling experiments, we isolate organisms and organic materials from the environments in which they occur in harmony with their complex surroundings. This entails increasing the size of the specimens from a Petri-dish scale to a fully functional on-site beehive structure, which alters the length and time scales in which mycelial fungi might act as a symbiotic agent for honeybee and vice versa. For example, we do not know if the antibacterial capacity of mycelia can inhibit the dispersal of beneficial bacteria in the honeybee nest microbiome. To effectively map the beneficial bioactivities of mycelia via designing its scaffolds, various compositions with various fungal species, growth substrates, and morphologies should be rigorously tested in both laboratory and field conditions. To grow many identical repetitions of mycelial parts and full-scale beehive prototypes for such experiments, fungal biofabrication processes should be improved to be more efficient in terms of human labour. However, even though some properties, such as surface qualities, bioactive agents, and densities may vary on different sizes and timescales, the primary goal of providing a good thermal environment for bees must be met. Therefore, the thermal dynamics of this coupled system in different hive morphologies, including heat distribution and water balance, are of particular interest to us.

We proposed two digital methods for generating nest morphologies. Devised specifically for fused deposition manufacturing, these methods aim to establish bespoke stay-in scaffolds for living mycelia and also for other biodesign narratives. As opposed to the commonly used casting technique where the nutrition substrate and mycelium culture are filled in a mould manually, by using fused deposition techniques to lay the mould as a stay-in scaffold with internal structure, we can radically increase the surface area within a given volume and encourage a more uniform distribution of hyphae within the composite material system. We continue to improve our digital design techniques in order to produce more versatile and efficient design models that facilitate the iterative and exploratory nature of designing with and for other living organisms. We further improve our digital design skills in order to create more flexible and efficient design models that support the iterative and experimental nature of designing with and for other living organisms. 

It is an intriguing challenge to develop a fungal bioproduction process and ensure its healthy maintenance as an integral part of another complex entity: the technologically enhanced beehive of HIVEOPOLIS [66]. According to the HIVEOPOLIS design brief, the fungal materiality and scaffold morphologies should synergistically support a variety of physical, mechanical, and chemical properties. First and foremost, a self-sustaining mycelial hive should have at least three mycelium growth qualities to ensure the bioavailability of fungal metabolites and enzymes, as well as their safety in a durable and warm hive structure: (1) maintained healthy mycelium colonisation for its biological activities like enzymes and beneficial volatiles production, (2) thick mycelial skin on spots where water protection is needed, and (3) aerial growth of hyphae in order to mechanically connect separate modules and towards the beehive interior for bees exposure to mycelium in its purer state. In conclusion, the idea of utilising mycelium materials as a living material, coevolving hosts in the nests of other creatures, including humans, is an intriguing novel concept that guides our research. Yet, the meaningful re-integration of other living entities into the bee habitats, and human indoor spaces, is a challenging but promising task. It requires economic, cultural, and technological positioning of biohybrid architectures in human society, and eventually all ecosystems of Earth, this also demands the cultivation of multispecies living narratives and practices in our everyday lives. For us, the challenge was to bring the performance of smaller scale prototypes and qualities of small-scale samples to full-scale prototypes, ready in time for honeybee field experiments which are highly season dependent. When the technical challenges are overcome, more research is needed, however, to discover the long-term events that mycelium may initiate as part of habitat architectures. The increased microbial diversity in terms of quantity—the number of distinct species—does not satisfy the goal to reach well-balanced microbial diversity. It is still speculative whether we will be able to effectively modulate the indoor microbiomes of our habitats or other organisms by incorporating dense mycelial networks into our architecture. We need to learn more about “who is there, where they live, and what they are doing” in our fungal designs. State of the art biotechnologies can aid in collecting this information in relation to our design probes. We can then use this knowledge in combination with the tools and methods developed in our fungal architecture community, to re-establish these causalities between geometrical, topological, and tactile aspects of our designs and microbial activities. In general, all these goals require focused groups of biodesign researchers collaborating with people in other scientific and engineering fields.

## Data Availability

Not applicable.

## References

[1] Last J.M. (2007). A Dictionary of Public Health.

[2] Semmelweis I.F. (1905). Semmelweis’ Gesammelte Werke.

[3] Pasteur L. On the Extension of the Germ Theory to the Etiology of Certain Common Diseases. http://ebooks.adelaide.edu.au/p/pasteur/louis/exgerm/.

[4] Ropars L., Dajoz I., Fontaine C., Geslin B. (2016). Which Impacts of Domesticated Honeybee Introductions and Management Practices on the Pollination Ecosystem Service in Urban Habitats?. https://www.researchgate.net/publication/309674183_Which_impacts_of_domesticated_honeybee_introductions_and_management_practices_on_the_pollination_ecosystem_service_in_urban_habitats.

[5] Rohlfs M., Churchill A.C.L. (2011). Fungal Secondary Metabolites as Modulators of Interactions with Insects and Other Arthropods. Fungal Genet. Biol..

[6] Janson E.M., Stireman J.O., Singer M.S., Abbot P. (2008). Phytophagous Insect–Microbe Mutualisms and Adaptive Evolutionary Diversification. Evolution.

[7] Biedermann P.H.W., Vega F.E. (2020). Ecology and Evolution of Insect–Fungus Mutualisms. Annu. Rev. Entomol..

[8] Davis T.S., Landolt P.J. (2013). A Survey of Insect Assemblages Responding to Volatiles from a Ubiquitous Fungus in an Agricultural Landscape. J. Chem. Ecol..

[9] Birkemoe T., Jacobsen R.M., Sverdrup-Thygeson A., Biedermann P.H.W., Ulyshen M.D. (2018). Insect-Fungus Interactions in Dead Wood Systems. Saproxylic Insects.

[10] Barcoto M.O., Carlos-Shanley C., Fan H., Ferro M., Nagamoto N.S., Bacci M., Currie C.R., Rodrigues A. (2020). Fungus-Growing Insects Host a Distinctive Microbiota Apparently Adapted to the Fungiculture Environment. Sci. Rep..

[11] Banham R. (1984). The Architecture of the Well-Tempered Environment.

[12] I’m Lost in Paris/R&Sie(n). https://www.archdaily.com/12212/im-lost-in-paris-rsien.

[13] Bat Access Brick. https://www.nhbs.com/bat-brick.

[14] Poikilohydric Living Walls Project. https://www.ucl.ac.uk/bartlett/architecture/about-us/innovation-enterprise/building-greener-cities-poikilohydric-living-walls.

[15] Seeley T.D., Kalett A., Zodrow K. (2019). The Lives of Bees, Untold Story of the Honeybee in Wild.

[16] Stokland J.N., Siitonen J., Jonsson B.G. (2012). Biodiversity in Dead Wood.

[17] Spencer-Booth Y. (1960). Feeding Pollen, Pollen Substitutes and Pollen Supplements to Honeybees. Bee World.

[18] Benevenute Parish J. (2019). Aspects of the Interactions between Honey Bees (*Apis mellifera*) and Propagules of Plant Pathogens. Ph.D. Thesis.

[19] Tihelka E. (2018). The Immunological Dependence of Plant-Feeding Animals on Their Host’s Medical Properties May Explain Part of Honey Bee Colony Losses. Arthropod-Plant Interact..

[20] Simone-Finstrom M., Evans J., Spivak M. (2009). Resin Collection and Social Immunity in Honey Bees. Evol. Int. J. Org. Evol..

[21] Anderson K., Sheehan T.H., Eckholm B., Mott B.M., Degrandi-Hoffman G. (2011). An Emerging Paradigm of Colony Health: Microbial Balance of the Honey Bee and Hive (*Apis mellifera*). Insectes Sociaux.

[22] Kostić A.Ž., Milinčić D.D., Petrović T.S., Krnjaja V.S., Stanojević S.P., Barać M.B., Tešić Ž.L., Pešić M.B. (2019). Mycotoxins and Mycotoxin Producing Fungi in Pollen: Review. Toxins.

[23] Free Living Bees Bees Don’t Make It Honey Team Do. https://www.freelivingbees.com/post/bees-don-t-make-honey-teams-do.

[24] Turner J.S. (2000). The Soul of the Superorganism. The Extended Organism: The Physiology of Animal-Built Structures.

[25] Thenius R., Schmickl T., Crailsheim K. (2008). Optimisation of a Honeybee-Colony’s Energetics via Social Learning Based on Queuing Delays. Connect. Sci..

[26] Schmickl T., Thenius R., Crailsheim K. (2012). Swarm-Intelligent Foraging in Honeybees: Benefits and Costs of Task-Partitioning and Environmental Fluctuations. Neural Comput. Appl..

[27] Zahadat P., Hahshold S., Thenius R., Crailsheim K., Schmickl T. (2015). From Honeybees to Robots and Back: Division of Labour Based on Partitioning Social Inhibition. Bioinspir. Biomim..

[28] Maes A.M. ElbBienen in Hamburg, Art in Public Space. https://annemariemaes.net/presentations/bee-laboratory-presentations-2/2019-elbbienen-in-hamburg-machine-art-in-public-space/.

[29] Thompson G. (2022). A Mycelium Hive—Growing Your Own. Bee Craft: The Independent Voice of British Beekeeping.

[30] Turner T. A Buzzworthy Beehive|Yanko Design. https://www.yankodesign.com/2017/04/07/a-buzzworthy-beehive/.

[31] Jones M., Mautner A., Luenco S., Bismarck A., John S. (2020). Engineered Mycelium Composite Construction Materials from Fungal Biorefineries: A Critical Review. Mater. Des..

[32] Jones M., Gandia A., John S., Bismarck A. (2021). Leather-like Material Biofabrication Using Fungi. Nat. Sustain..

[33] Mazur R. (2015). Mechanical Properties of Sheets Comprised of Mycelium: A Paper Engineering Perspective. Honors Thesis.

[34] Su C.-H., Liu S.-H., Yu S.-Y., Hsieh Y.-L., Ho H.-O., Hu C.-H., Sheu M.-T. (2005). Development of Fungal Mycelia as a Skin Substitute: Characterization of Keratinocyte Proliferation and Matrix Metalloproteinase Expression during Improvement in the Wound-Healing Process. J. Biomed. Mater. Res. A.

[35] Antinori M.E., Contardi M., Suarato G., Armirotti A., Bertorelli R., Mancini G., Debellis D., Athanassiou A. (2021). Advanced Mycelium Materials as Potential Self-Growing Biomedical Scaffolds. Sci. Rep..

[36] Xing Y., Brewer M., El-Gharabawy H., Griffith G., Jones P. (2018). Growing and Testing Mycelium Bricks as Building Insulation Materials. IOP Conf. Ser. Earth Environ. Sci..

[37] Girometta C., Picco A.M., Baiguera R.M., Dondi D., Babbini S., Cartabia M., Pellegrini M., Savino E. (2019). Physico-Mechanical and Thermodynamic Properties of Mycelium-Based Biocomposites: A Review. Sustainability.

[38] Pelletier M.G., Holt G.A., Wanjura J.D., Bayer E., McIntyre G. (2013). An Evaluation Study of Mycelium Based Acoustic Absorbers Grown on Agricultural By-Product Substrates. Ind. Crops Prod..

[39] Holt G.A., Mcintyre G., Flagg D., Bayer E., Wanjura J.D., Pelletier M.G. (2012). Fungal Mycelium and Cotton Plant Materials in the Manufacture of Biodegradable Molded Packaging Material: Evaluation Study of Select Blends of Cotton Byproducts. J. Biobased Mater. Bioenergy.

[40] Appels F.V.W., Camere S., Montalti M., Karana E., Jansen K.M.B., Dijksterhuis J., Krijgsheld P., Wösten H.A.B. (2019). Fabrication Factors Influencing Mechanical, Moisture- and Water-Related Properties of Mycelium-Based Composites. Mater. Des..

[41] Van Wylick A., Monclaro A.V., Elsacker E., Vandelook S., Rahier H., De Laet L., Cannella D., Peeters E. (2021). A Review on the Potential of Filamentous Fungi for Microbial Self-Healing of Concrete. Fungal Biol. Biotechnol..

[42] Grown.bio Grow It Yourself Mycelium Packaging Kit. https://www.grown.bio/wp-content/uploads/2020/07/GIY_Manual_GrownBio.pdf.

[43] Mycelium Textile. https://neffa.nl/portfolio/mycelium-textile/.

[44] Adamatzky A., Gandia A., Ayres P., Wösten H., Tegelaar M. Adaptive Fungal Architectures. http://links-series.com/wp-content/revues/5-6-Proust/10-Adamatsky-Gandia-Ayresetal-Adaptive_fungal_architectures-LINKs_serie_5_6.pdf.

[45] Alima N., Snooks R., McCormack J. (2022). Bio Scaffolds: The Orchestration of Biological Growth through Robotic Intervention. Int. J. Intell. Robot. Appl..

[46] Blast Studio 3D Prints Column from Mycelium to Make “Architecture that Could Feed People”. https://www.dezeen.com/2022/01/18/blast-studio-tree-column-mycelium-design/.

[47] Goidea A., Andreen D., Floudas D. (2020). Pulp Faction: 3d Printed Material Assemblies through Microbial Biotransformation. Proceedings of Fabricate 2020: Making Resilient Architecture.

[48] Mitchell D. (2016). Ratios of Colony Mass to Thermal Conductance of Tree and Man-Made Nest Enclosures of *Apis mellifera*: Implications for Survival, Clustering, Humidity Regulation and Varroa Destructor. Int. J. Biometeorol..

[49] Stamets P.E., Naeger N.L., Evans J.D., Han J.O., Hopkins B.K., Lopez D., Moershel H.M., Nally R., Sumerlin D., Taylor A.W. (2018). Extracts of Polypore Mushroom Mycelia Reduce Viruses in Honey Bees. Sci. Rep..

[50] Evans J.D., Schwarz R.S. (2011). Bees Brought to Their Knees: Microbes Affecting Honey Bee Health. Trends Microbiol..

[51] Reinbacher L., Fernandez Ferrari C., Angeli S., Schausberger P. (2018). Effects of Metarhizium Anisopliae on Host Choice of the Bee-Parasitic Mite Varroa Destructor. Acarologia.

[52] Han J.O., Naeger N.L., Hopkins B.K., Sumerlin D., Stamets P.E., Carris L.M., Sheppard W.S. (2021). Directed Evolution of Metarhizium Fungus Improves Its Biocontrol Efficacy against Varroa Mites in Honey Bee Colonies. Sci. Rep..

[53] Türkekul İ., Gülmez Y. (2016). Propolis: An Enrichment Material for Mycelium Development of Oyster Mushroom (*Pleurotus Ostreatus*). Nat. Resour..

[54] Oxman N. Digital Craft: Fabrication Based Design in the Age of Digital Production. Proceedings of the 2007 International Conference on Ubiquitous Computing.

[55] https://github.com/johnharding/Biomorpher.

[56] Grasshopper3D Is a Visual Programming Language (VPL) Platform for Use in the CAD Package Rhinoceros3D. https://www.rhino3d.com/download/grasshopper/1.0/wip/rc.

[57] Takagi H. (2001). Interactive Evolutionary Computation: Fusion of the Capabilities of EC Optimization and Human Evaluation. Proc. IEEE.

[58] Harding J., Brandt-Olsen C. (2018). Biomorpher: Interactive Evolution for Parametric Design. Int. J. Archit. Comput..

[59] Seeley T., Morse R. (1976). The Nest of the Honey Bee (*Apis mellifera* L.). Insectes Sociaux.

[60] https://www.grasshopper3d.com/group/millipede.

[61] Ilgun A., Ayres P. (2016). Co-Occupied Architectural Boundaries as 3D Printed Scaffolds Embellished Through Self-Organised Construction.

[62] 3d Printing. Kai Parthy Introduces GROWLAY Indoor Farming Material. https://3dprinting.com/news/german-filament-creator-introduces-indoor-farming-material/.

[63] https://shop.keramik.at/c/59/a/20116/ueber-1250%C2%B0C/Sio2-Paperclay-PCLI-weiss-125-kg-Pkg-.html.

[64] Stamets P. (1993). Growing Gourmet and Medicinal Mushrooms.

[65] OSBeehives Source Files. https://www.osbeehives.com.

[66] Ilgün A., Angelov K., Stefanec M., Schönwetter-Fuchs S., Stokanic V., Vollmann J., Hofstadler D.N., Kärcher M.H., Mellmann H., Taliaronak V. (2021). Bio-Hybrid Systems for Ecosystem Level Effects.

